# Role of blood metabolites in mediating the effect of gut microbiota on chronic gastritis

**DOI:** 10.1128/spectrum.01490-24

**Published:** 2024-10-15

**Authors:** Tianying Liu, Zhian Chen, Li Sun, Lihui Xiong

**Affiliations:** 1 College of Basic Medical Sciences, Changchun University of Traditional Medicine, Changchun, China; 2 College of Integrative Medicine, Changchun University of Traditional Medicine, Changchun, China; 3 Jilin Academy of Chinese Medical Sciences, Changchun, China; 4 Changchun University of Traditional Medicine, Changchun, China; Tainan Hospital, Ministry of Health and Welfare, Tainan, Taiwan

**Keywords:** Mendelian randomization analysis, gut microbiota, blood metabolites, chronic gastritis, mediating effect

## Abstract

Exploring the link between gut microbiota and chronic gastritis (CG), and assessing the potential mediating influence of blood metabolites. Using aggregated data from genome-wide association studies (GWAS), we performed a two-sample Mendelian randomization (MR) analysis to explore the genetic links between gut microbiota (412 types) and CG (623,822 cases). Furthermore, we utilized a two-step MR approach to measure the extent to which blood metabolites (1,400 types) mediate the impact of gut microbiota on CG. Through MR, we identified that three genetically predicted gut microbiota increased the risk of CG: the ubiquinol-8 biosynthesis pathway (OR 1.149, 95%CI 1.022–1.291), *Odoribacter* from the Porphyromonadaceae family (OR 1.260, 95%CI 1.044–1.523), and *Coprococcus* from the Lachnospiraceae family (OR 1.125, 95%CI 1.010–1.253). Currently, there is no evidence to suggest that genetically predicted CG affects the risk of gut microbiota. Four blood metabolites mediated the proportionate changes in genetically predicted gut microbiota: levels of 4-hydroxyphenylacetate levels by 14.9% (95% CI −0.559%, 30.3%), palmitoleate (16:1n7) levels, and the phosphate to alanine ratio together mediated the same microbiota by 6.97% (95% CI −1.61%, 15.6%) and 7.91% (95% CI −1.67%, 17.5%), while the phosphate to alanine ratio and X-12839 levels together mediated the same microbiota by 8.48% (95% CI −2.87%, 19.8%) and 10.7% (95% CI 0.353%, 21.1%). In conclusion, our research has confirmed a causal link between gut microbiota, blood metabolites, and CG. Metabolites such as 4-hydroxyphenylacetate levels, palmitoleate (16:1n7) levels, the phosphate to alanine ratio, and X-12839 levels have relatively significant mediating roles between gut microbiota and CG. These metabolites may influence the occurrence and development of CG by regulating inflammatory responses, energy metabolism, and gut barrier function. However, the majority of the influence of gut microbiota on CG remains unclear, necessitating further research into other potential mediating risk factors. Clinically, it is crucial to focus on patients suffering from CG who exhibit dysbiosis of gut microbiota.

**IMPORTANCE:**

The results indicate that interactions between particular gut microbiota and blood metabolites may significantly contribute to the onset and progression of CG. These findings offer new insights and potential targets for early diagnosis, personalized treatment, and prevention of CG.

## INTRODUCTION

Chronic gastritis (CG) is characterized by diverse chronic inflammatory lesions in the gastric mucosa, each resulting from distinct etiologies. It is a common condition, usually presenting with no obvious symptoms or experiencing stomach pain and bloating. It leads to the incidence of gastric diseases, affecting over 4.4 billion people globally (1). As economies rapidly develop, significant changes in the lifestyles, dietary habits, and daily routines of young people have contributed to a rise in CG cases within this group (2). Pediatric assessments and analyses have shown a notable rise in the occurrence of mild CG and non-specific gastritis within our population over the last 10 years (3). Chronic persistent inflammation is widely regarded as a trigger for cancer; chronic atrophic gastritis (CAG) is a critical phase in the inflammatory transformation process of tumors, and the currently accepted model of Gastric cancer (GC) progression follows the sequence: normal gastric mucosa → superficial gastritis → atrophic gastritis → intestinal metaplasia → dysplasia → gastric cancer (4–6). Therefore, early prevention of CG is crucial.

The gut microbiota is highly dynamic, and its imbalance can impact CG patients in various ways, such as exacerbating gastritis symptoms (acid reflux, nausea) and triggering or worsening other digestive system diseases (like irritable bowel syndrome) (7). Numerous animal experiments have shown a close link between CG and gut microbiota (8–11). An observational study on children further indicated that gastritis might lead to changes in the fecal microbiota composition (12). Interestingly, an MR analysis of Chinese genes demonstrated a strong causal relationship between blood metabolites and gut microbiota (13). This relationship not only regulates gastrointestinal diseases but also plays a preventive role in various metabolic diseases, such as cardiovascular diseases and diabetes (13–15). However, this relationship lacks corresponding animal experimental studies. Additionally, current research on blood metabolites and gastrointestinal diseases primarily focuses on using metabolomics to study GC or other directions, with few studies specifically addressing CG. Epidemiological and animal studies may have uncontrollable confounding factors, human errors, environmental impacts, and missing variables, which can ultimately lead to biased results. It is crucial to minimize or avoid these confounding factors in study design. Therefore, it is essential to determine the relationship between blood metabolites and CG, as well as to clarify their mediating role in gut microbiota-CG.

Mendelian randomization serves as a research technique to investigate potential causal connections, utilizing genetic variations as instrumental variables (IVs) to assess the impact of exposure factors on health outcomes (16, 17). This approach, grounded in the principles of Mendelian genetics, effectively reduces confounding factors, prevents reverse causation, and aids in evaluating long-term effects. Specifically, our study employed Mendelian mediation analysis techniques, which can test and quantify the role of one or more mediating variables between genetic variations (as exposure) and health outcomes. This not only reveals potential causal pathways between exposure and outcomes but also provides new insights into how intervention in the mediation process can address CG. Herein, we conducted a comprehensive MR analysis using a large database to determine whether there is a causal relationship between blood metabolites and CG, their mediating role between the gut microbiome and CG, and to reveal the complex interconnections among the gut microbiome, blood metabolites, and CG.

## MATERIALS AND METHODS

### Study design

The data set for our study was obtained from public databases and previously received approval from the Institutional Review Board (IRB) for related studies. Therefore, no additional approvals were needed for this analysis. We have presented all study results in detail in the article and its appendices.

In our research, we employed a bidirectional, two-sample MR method to investigate the reciprocal causal relationships between gut microbiota and CG (17). Single-nucleotide polymorphisms (SNPs) served as IVs for this analysis (17). MR operates under three key assumptions: (i) the chosen genetic variants need to have a strong association with the exposure; (ii) the link between genetic variants and the outcomes is mediated solely by the exposure; (iii) genetic variants do not correlate with any potential confounders (18). Additionally, the mediation role of 1,400 blood metabolites in Mendelian mediation effect outcomes was also studied.

### Instrumental variable selection

In our study, we first selected SNPs at a genome-wide significance level of *P* = 1 × 10^−5^ (19). Next, based on the estimated values from the European population of the 1,000 Genomes Project, these SNPs were filtered through linkage disequilibrium (LD) clustering, setting a clustering window of 10,000 kb and an r^2^ of 0.001 (20). If SNPs for a specific exposure factor were missing in the outcome data set, proxy SNPs marked for linkage disequilibrium were used. After removing linkage disequilibrium, 4,048 SNPs remained as IVs for gut microbiota, 33,571 SNPs for metabolites, and 37 SNPs for CG. The *F*-statistic is derived from the proportion of variance that SNPs account for, with the formula: *F* = *R*^2^ (*N − K* − 1)/[(1 *R*^2^) *k*], where *K* is the number of IVs, *N* is the total sample size, *R*^²^ is the proportion of the exposure variable explained by the IVs (explained variance), and 1 *R*^2^ is the proportion of variance unexplained by the IVs (21, 22). Finally, we excluded weak IVs with an *F*-statistic less than 10.

### Gut microbiota data sources

Summary statistics for gut microbiota are derived from a published GWAS meta-analysis (23). All data can be accessed from the NHGRI-EBI GWAS directory at http://ftp.ebi.ac.uk/pub/databases/gwas/summary_statistics/GCST90027001-GCST90028000/. The accession numbers for the gut microbiome data are GCST90027446-GCST90027857.Or directly downloaded from https://dutchmicrobiomeproject.molgeniscloud.org (23). The GWAS includes 207 taxa and 205 pathways.

### Blood metabolites data sources

All the data utilized in our study are accessible to the public (17, 24). The GWAS includes up to 1,400 individuals of European descent, with genome-wide associations covering 1,091 blood metabolites and 309 metabolites. Summary statistics for the GWAS are stored in the GWAS Catalog (https://www.ebi.ac.uk/gwas/). Accession numbers for European GWAS: GCST90199621-90201020; accession numbers for non-European GWAS: GCST90201021-90204063.

### Chronic gastritis data sources

GWAS summary statistics on CG involved 3,645 cases from European ancestry, 441,451 European ancestry control cases, 744 cases of East Asian ancestry, and 177,982 East Asian ancestry control cases. After quality filtering and statistical estimation, a total of approximately 2.418 million genetic variants were analyzed. These data can be accessed via the IEU OpenGWAS project at https://gwas.mrcieu.ac.uk/datasets/ebi-a-GCST90018825/, or through the GWAS Catalog (https://www.ebi.ac.uk/gwas/studies/GCST90018825).

### Statistical analysis

In this study, we utilized R software (version 4.3.1, http://www.r-project.org) and the “TwoSampleMR” package (version 0.5.6) to conduct MR analyses (17). To handle MR pleiotropy residual sum and outlier (MR-PRESSO), we employed the “MRPRESSO” package in R. Calculations for the robust adjusted profile score (MR.RAPS) were conducted with the “MR.RAPS” package. Additionally, we used the mRnd tool (https://cnsgenomics.shinyapps.io/mRnd/) to calculate the statistical power for MR. We also used the PhenoScanner tool (www.phenoscanner.medschl.cam.ac.uk) to evaluate all known phenotypes associated with the genetic instruments used in this analysis (25).

### Primary analysis

**Fig 1:**
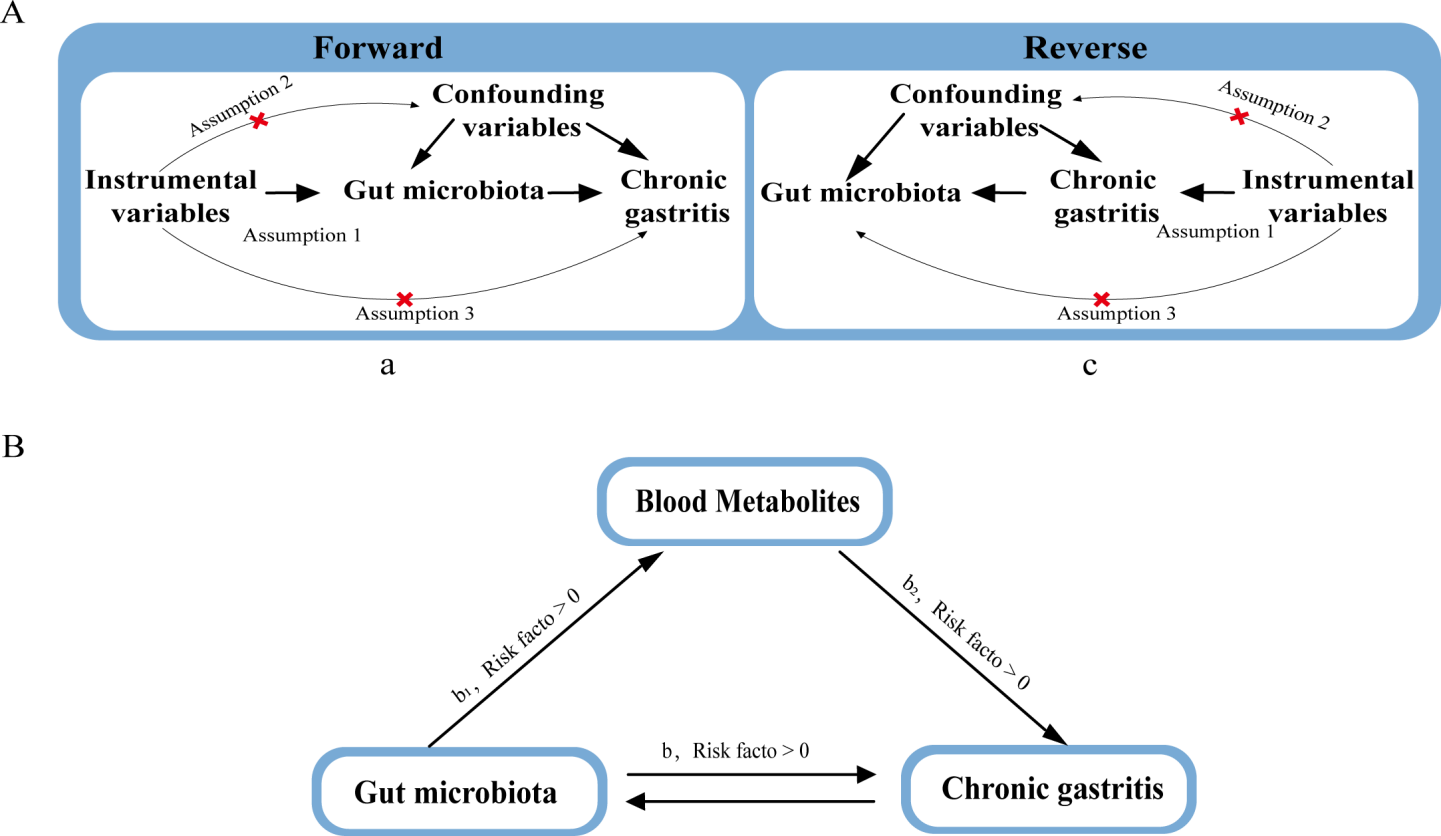
Diagrams depicting the relationships analyzed in this research. (**A**) The overall impact between Gut microbiota and CG, with a representing the total impact with Gut microbiota as the predictor and CG as the result, and c representing the total impact with CG as the predictor and Gut microbiota as the result. (**B**) The overall impact was broken down into (i) an indirect impact using a two-step approach (where *a* is the total impact of gut microbiota on blood metabolites, and *b*_2_ is the impact of blood metabolites on CG) along with the product method (*b*_1_ × *b*_2_) and (ii) a direct impact (*b* = *a* – *b*_1_ × *b*_2_). The proportion mediated was calculated as the indirect impact divided by the total impact.

Figure 1 illustrates the brief workflow of the analysis. Initially, a bidirectional two-sample MR was conducted to assess the mutual causal relationships between gut microbiota and CG, termed the overall effect.

Inverse variance weighting (IVW) utilizes variances estimated by a specific statistical model to assign weights to each SNP in the composite outcome, enhancing the precision and reliability of the overall estimate (26). Cochran’s Qs, along with its corresponding *P*-value, evaluates heterogeneity across SNPs. Subsequently, MR-Egger, weighted median, and MR-PRESSO analyses were used to test the robustness of associations and detect potential pleiotropy. Additionally, Bayesian weighted methods were employed to validate data. Different approaches were utilized to derive MR estimates based on varying validity assumptions. MR-Egger is used to detect and adjust for non-ideal aspects of IVs with the intercept term used to detect and correct biases caused by direct effects. As long as over 50% of the IVs are valid, the weighted median can effectively reduce biases, enhancing the reliability of causal inference (27). Pleiotropy residual sum and outlier analysis in MR research can detect and correct biases and errors that might affect causal inferences, thereby improving the reliability of MR analyses and making the conclusions more robust and credible. Bayesian validation also provides consistent estimates when dealing with complex data and uncertainties.

### Mediation analysis

We also performed a mediation analysis through a two-step MR approach to investigate if metabolites serve as mediators in the causal pathway from gut microbiota to CG outcomes (Fig. 1B) (17). The total influence can be divided into indirect effects (mediated via an intermediary) and direct effects (not mediated through an intermediary) (17). The overall influence of gut microbiota on CG is decomposed into (i) the direct effect of gut microbiota on CG, i.e., the overall effect (b in Fig. 1B) and (ii) the mediated effect of gut microbiota through an intermediary (*b*1 × *b*2 in Fig. 1B). The direct effect is calculated as the total effect minus the mediated effect. When both the total effect and mediated effect align in the same direction, it indicates a mediation effect. The calculation for the mediation percentage is given by the ratio: mediated effect/total effect (28). Additionally, 95% confidence interval is calculated using the delta method.

### Sensitivity analysis

Sensitivity analysis is a crucial step in MR studies, employed to evaluate the robustness and reliability of the findings (29). The MR Steiger filtering test preprocesses the data, selecting SNPs that have a stronger explanatory power for the exposure and removing those that may directly influence the outcomes. This method helps enhance the overall quality of the analysis and the interpretability of the results.

Cochran’s Q statistic along with funnel plots assess heterogeneity within SNPs. Simultaneously, MR-Egger intercept approach and MR-PRESSO method are applied to identify horizontal pleiotropy. MR-Egger intercept method serves as the basis for bias detection and correction. When outliers may be present in the data, robustness is tested using the weighted median and leave-one-out analysis. MR-PRESSO is specifically applied to handle anomalous data points. Finally, leave-one-out analysis is employed to identify any individual IVs (SNPs) that disproportionately affect the overall causal estimate.

## RESULTS

Based on the selection criteria for IVs, we chose 49, 99, and 52 SNPs as IVs for gut microbiota, blood metabolites, and CG, respectively. In the univariate MR analysis, all IVs had *F*-statistics over 10, indicating a low risk of bias due to weak instruments (30). Our study provides a 100% confidence level in detecting the causal impact of gut microbiota on CG risk.

### Association of gut microbiota with CG

**Fig 2:**
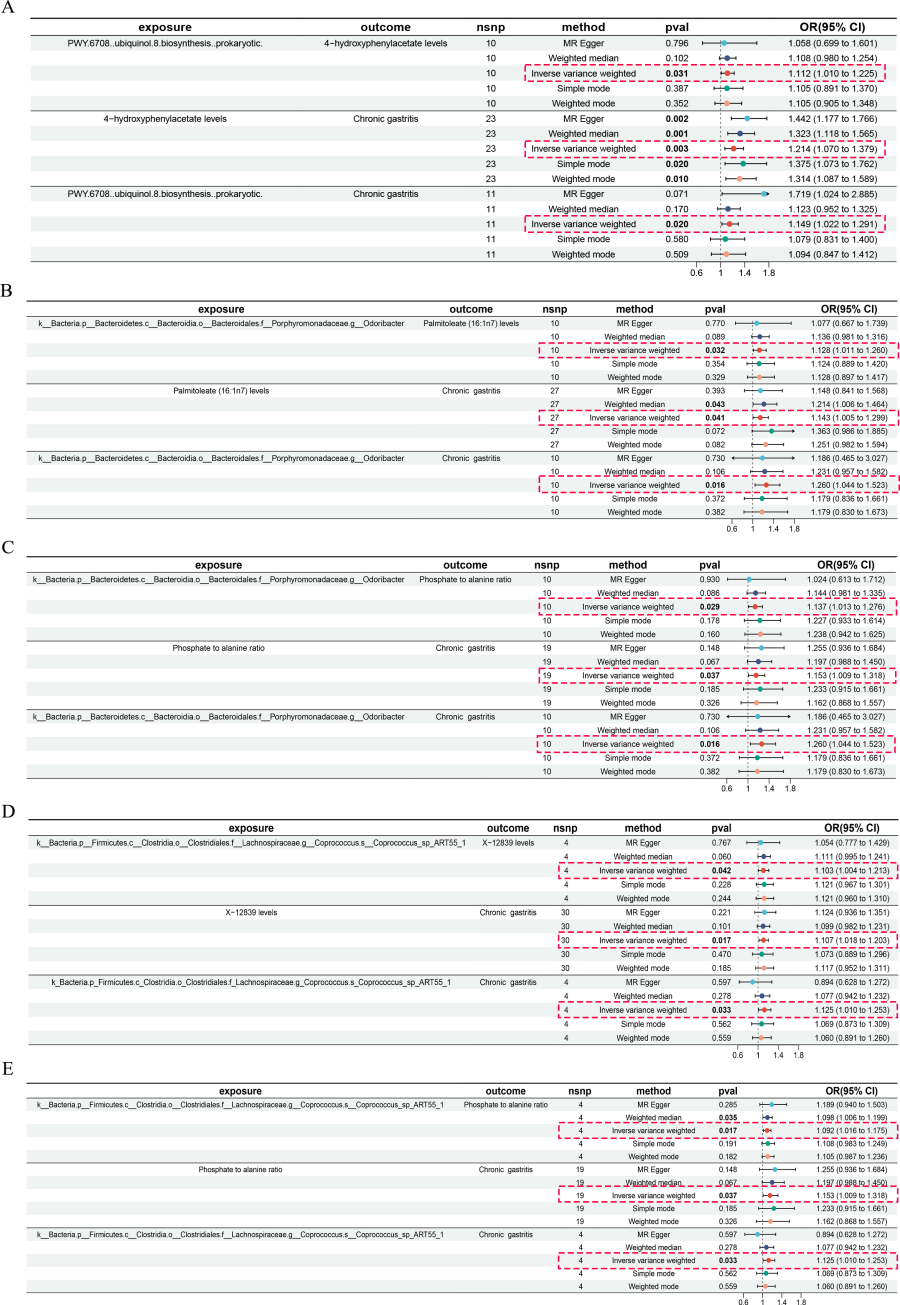
Graph of risk factor data for all samples.

**Fig 3:**
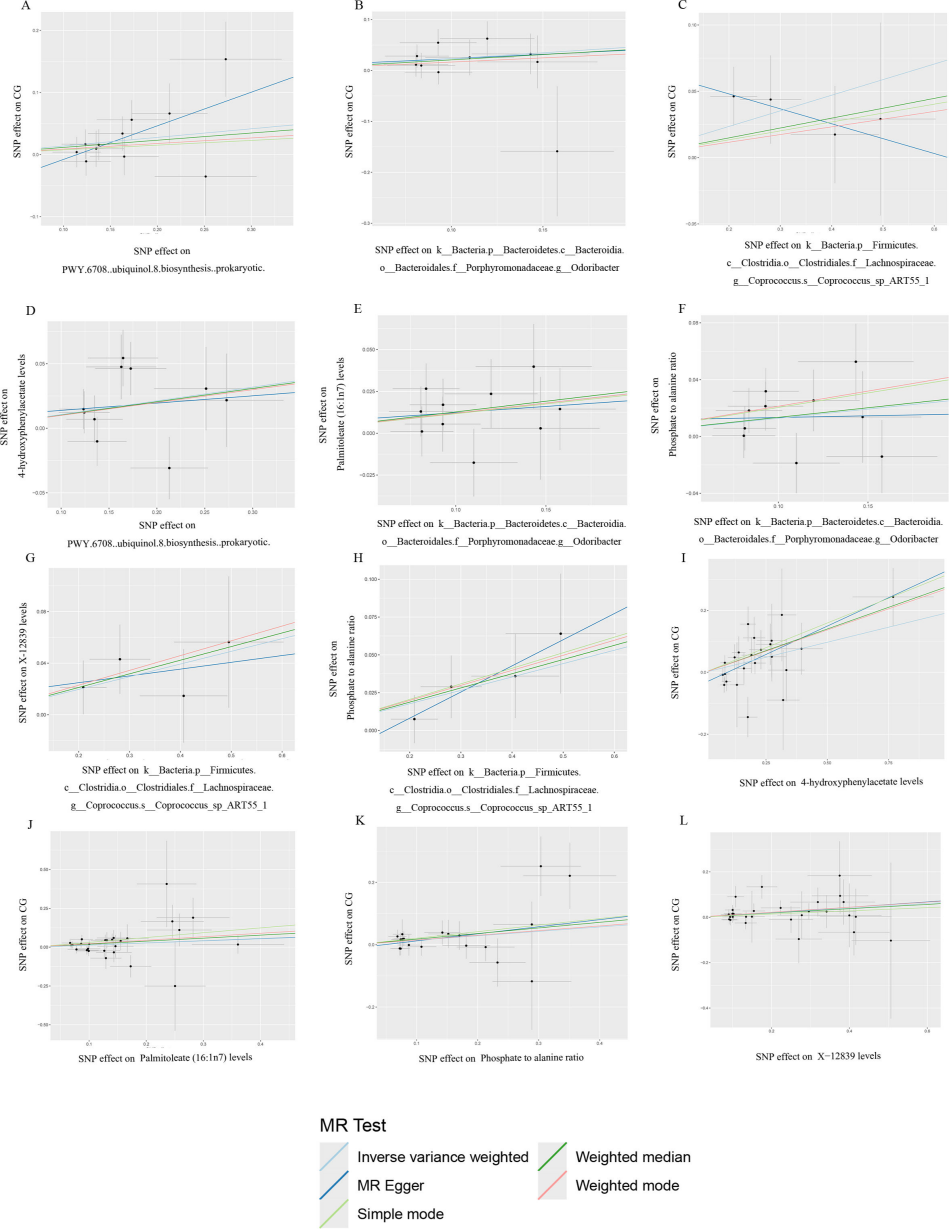
Scatter plot of all positive results.

**Fig 4:**
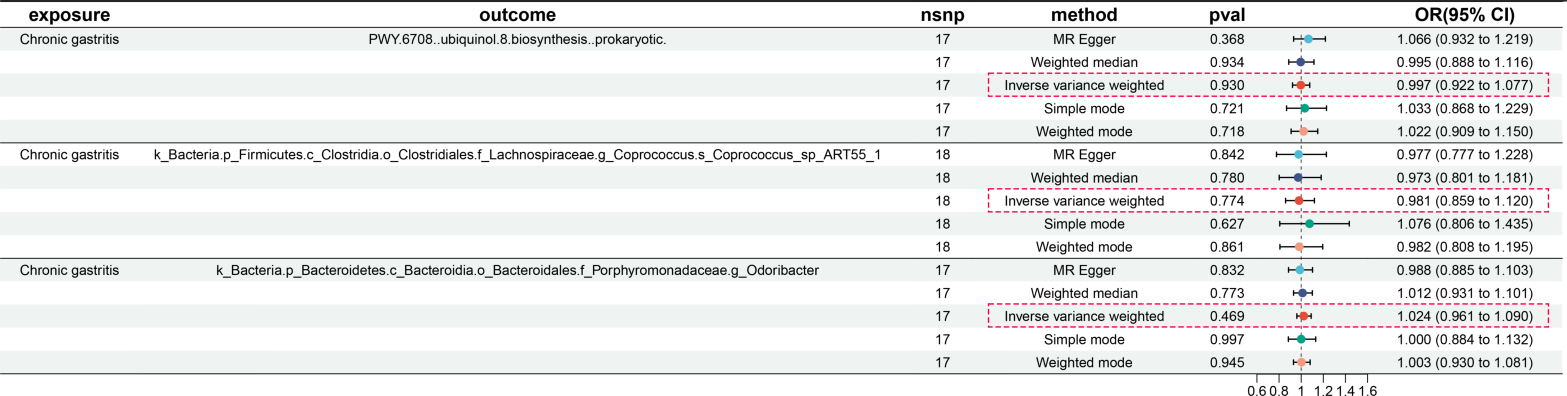
Reverse causality between CG and three gut microbiota.

IVW, MR-Egger, and weighted median provide different statistical strategies to infer potential causal relationships between gut microbiota and CG from genetic data. After analyzing with these three methods, we found a positive correlation between gut microbiota and CG, identifying three types of bacteria that pose a risk to CG (Fig. 2 and 3). For PWY.6708..ubiquinol.8.biosynthesis..prokaryotic, per SD increase: IVW OR = 1.149, 95% CI 1.022–1.291, *P* = 0.020. For k__Bacteria.p__Bacteroidetes.c__Bacteroidia.o__Bacteroidales.f__Porphyromonadaceae.g__*Odoribacter*, per SD increase: IVW OR = 1.260, 95% CI 1.044–1.523, *P* = 0.016. For k_Bacteria.p_Firmicutes.c_Clostridia.o_Clostridiales.f_Lachnospiraceae.g_Coprococcus.s_*Coprococcus*_sp_ART55_1, per SD increase: IVW OR = 1.125, 95% CI 1.010–1.253, *P* = 0.033. However, our MR analysis showed no reverse causal relationship between genetically predicted CG and these three gut microbiota (i.e., no causal relationship from genetically predicted gut microbiota to CG). Reverse analysis for PWY.6708..ubiquinol.8.biosynthesis..prokaryotic with IVW showed OR = 0.997, 95% CI 0.922–1.077, *P* = 0.930; for k__Bacteria.p__Bacteroidetes.c__Bacteroidia.o__Bacteroidales.f__Porphyromonadaceae.g__*Odoribacter*, IVW OR = 1.024, 95% CI 0.961–1.090, *P* = 0.469; and for k_Bacteria.p_Firmicutes.c_Clostridia.o_Clostridiales.f_Lachnospiraceae.g_Coprococcus.s_*Coprococcus*_sp_ART55_1, IVW OR = 0.981, 95% CI 0.859–1.120, *P* = 0.774. Results are shown in Fig. 4.

### Association of gut microbiota with blood metabolites

**Fig 5:**
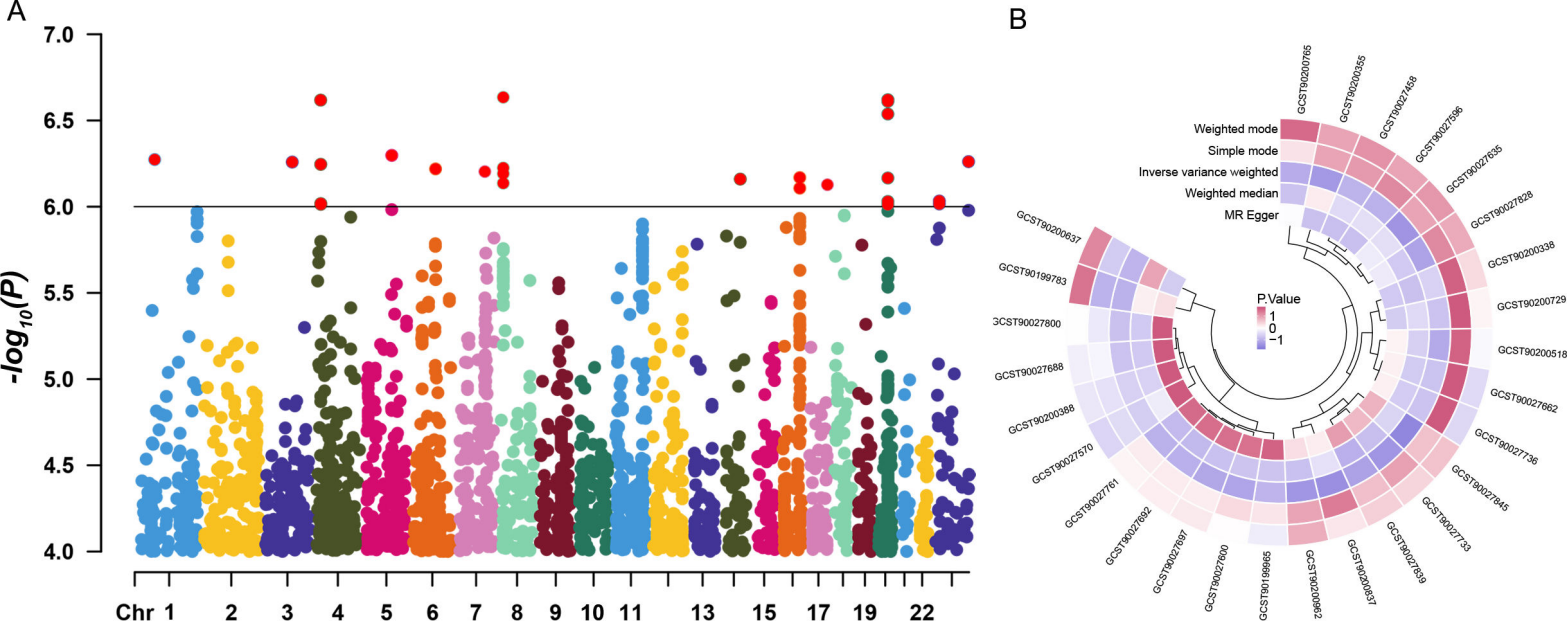
Circular numerical heatmap and Manhattan plot of gut microbiota and metabolite risk factors. Each point in the figure represents an SNP, with red points indicating metabolites that showed significant associations in our analysis. The smaller the *P*-value of a genetic locus, the stronger its association with phenotypic traits or diseases.

Utilizing the IVW, MR-Egger, and weighted median methods, a positive correlation was found between genetically predicted gut microbiota and blood metabolites risks, identifying three types of microbiota that regulate four blood metabolites as risk factors (Fig. 2, 3 and 5). The microbiota PWY.6708..ubiquinol.8.biosynthesis..prokaryotic controls the blood metabolite 4-hydroxyphenylacetate levels: IVW (OR = 1.112, 95% CI 1.010–1.225, *P* = 0.031). Microbiota k__Bacteria.p__Bacteroidetes.c__Bacteroidia.o__Bacteroidales.f__Porphyromonadaceae.g__*Odoribacter* controls blood metabolite palmitoleate (16:1n7) levels: IVW (OR = 1.128, 95% CI 1.011–1.260, *P* = 0.032). The same microbiota also controls phosphate to alanine ratio: IVW (OR = 1.137, 95% CI 1.013–1.276, *P* = 0.029). Microbiota k__Bacteria.p__Firmicutes.c__Clostridia.o__Clostridiales.f__Lachnospiraceae.g__Coprococcus.s_*Coprococcus*_sp_ART55_1 controls metabolite X-12839 levels: IVW (OR = 1.103, 95% CI 1.004–1.213, *P* = 0.042). Additionally, this microbiota also controls phosphateto alanine ratio: IVW (OR = 1.092, 95% CI 1.016–1.175, *P* = 0.017). Each ID corresponds to a specific enrichment analysis result (Tables S1 and S2).

### Association of blood metabolites with CG

Using blood metabolites as the exposure and CG as the outcome, the risk factor results are shown in Fig. 2, and the scatter plot can be seen in Fig. 3. Figure 5 displays the outcomes of the cyclic numerical thermograms. Genetically predicted blood metabolites showed a significant positive correlation with CG, identifying four risk factors among the blood metabolites. The metabolite 4-hydroxyphenylacetate levels were analyzed using IVW (OR = 1.214, 95% CI 1.070–1.379, *P* = 0.003). The metabolite palmitoleate (16:1n7) levels were found using IVW (OR = 1.143, 95% CI 1.005–1.299, *P* = 0.041). The metabolite phosphate to alanine ratio was analyzed using IVW (OR = 1.153, 95% CI 1.009–1.318, *P* = 0.037). The metabolite X-12839 levels were determined using IVW (OR = 1.107, 95% CI 1.018–1.203, *P* = 0.017). Estimates from IVW, MR-Egger, and weighted median methods showed consistency in their directional estimates.

### Percentage of the relationship between gut microbiota and CG mediated by blood metabolites

**Fig 6:**
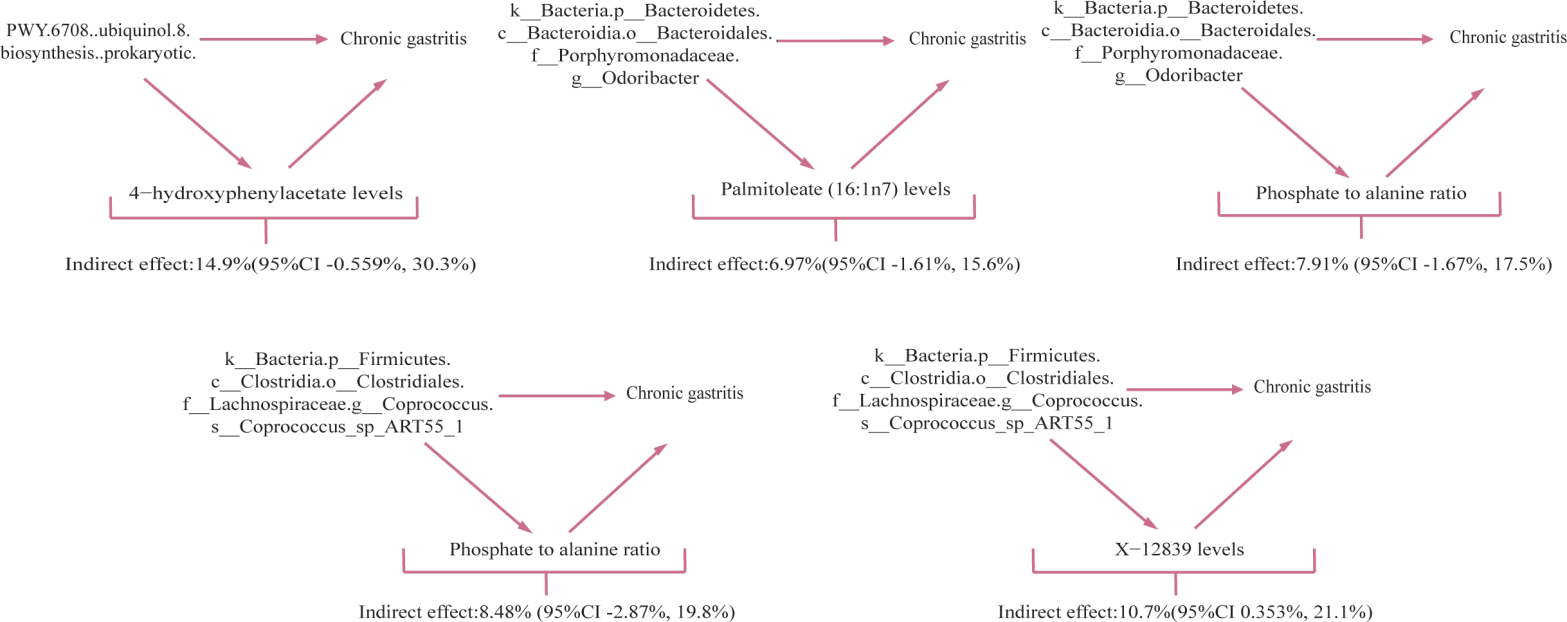
Schematic representation of the mediating effects of the four blood metabolites.

Our research analyzed four metabolites as mediators in the pathway from gut microbiota to CG. The results show that an increase in these four metabolites is associated with an elevated risk of CG, which is linked to the gut microbiota. As shown in Fig. 2 and 6, our study indicates that the metabolite 4-hydroxyphenylacetate levels mediate 14.9% (95% CI −0.559%, 30.3%) of the CG risk increase related to the microbiota PWY.6708..ubiquinol.8.biosynthesis..prokaryotic. The metabolites palmitoleate (16:1n7) levels and phosphate to alanine ratio jointly mediate 6.97% (95% CI −1.61%, 15.6%) and 7.91% (95%CI −1.67%, 17.5%) of the CG risk increase associated with the microbiota k__Bacteria.p__Bacteroidetes.c__Bacteroidia.o__Bacteroidales.f__Porphyromonadaceae.g__*Odoribacter*. Additionally, the metabolites phosphate to alanine ratio and X-12839 levels jointly mediate 8.48% (95% CI −2.87%, 19.8%) and 10.7% (95% CI 0.353%, 21.1%) of the CG risk increase associated with the microbiota k__Bacteria.p__Firmicutes.c__Clostridia.o__Clostridiales.f__Lachnospiraceae.g__Coprococcus.s__*Coprococcus*_sp_ART55_1.

### Sensitivity analysis

We conducted multiple sensitivity analyses to detect and correct for potential pleiotropy in our causal estimates. Cochran’s Q test and funnel plots demonstrated no signs of heterogeneity or asymmetry across SNPs, confirming the consistency of the causal link (17). In our study, the MR-Egger intercept provided slight indications of pleiotropy. Through leave-one-out analysis, we verified the contribution of each SNP to the overall causal estimate and systematically reconducted the MR analysis after excluding each SNP. The consistency of the results further confirmed the significant contribution of all SNPs to the causative link.

## DISCUSSION

Recent researches have highlighted the connection between gut microbiota and CG (8–11). However, current research is often limited to the regulatory effects of certain drugs on CG or observational studies, which may be affected by human factors and confounders (31–35). Our study seeks to clarify the causal link between gut microbiota and CG. Based on existing GWAS data, we use MR analysis to explore the link between gut microbiota and CG and to determine if blood metabolites mediate this causal connection. Bidirectional two-sample MR results significantly associate gut microbiota with increased CG risk. Additionally, no reverse causality was detected between CG and gut microbiota. Two-step MR results indicate potential mediating roles for metabolites 4-hydroxyphenylacetate levels, palmitoleate (16:1n7) levels, phosphate to alanine ratio, and X-12839 levels in the link between gut microbiota and CG. Different MR analysis methods propose various assumptions regarding pleiotropy and weak IVs, yet our results are largely robust.

To date, our study is the inaugural use of MR to explore the causal link between gut microbiota, blood metabolites, and CG, identifying three gut microbiota associated with the risk of brain injury. Additionally, the study confirmed that four blood metabolites mediate the connection between the gut microbiome and CG (36–39). CG is the onset of GC, and our results provide a more detailed comparison than previous studies between the two. Clinical studies by Hanjing Li et al. (40) sequencing the 16S rRNA genes from 176 fecal samples in Fujian, China, showed significant differences in gut microbiota characteristics due to *Helicobacter pylori* infection among patients with chronic non-atrophic gastritis (CNAG) and CAG. Fuhao Chu et al. (41) demonstrated that persistent chronic inflammation can disrupt gut microbiota, alter fecal metabolomic profiles, and stimulate immune responses to control the levels of various inflammatory cytokines, thus hastening the development of precancerous lesions in gastric cancer (PLGC). However, these studies—one clinical and one animal—may be more prone to reverse causation or other potential confounders than MR analysis.

The gut microbiome is intricately linked with the host’s overall metabolic processes (42). Within the gastrointestinal system of healthy adults, *Firmicutes* is one of the most abundant groups, accounting for about 40%–60% of total gut bacteria, with *Bacteroidetes* typically similar in number, around 20%–40%, followed by *Actinobacteria*, *Proteobacteria*, and *Verrucomicrobia* (43, 44). The diversity and richness of these microbiota are crucial for maintaining intestinal health and play key roles in various physiological processes. However, the composition of gut microbiota differs among individuals, easily influenced by genetics, age, diet, lifestyle, and environment (45). Once the balance of the gut microbiota is disrupted (also known as dysbiosis), it may lead to the development of various diseases, such as obesity, type 2 diabetes, inflammatory bowel disease, irritable bowel syndrome, cardiovascular diseases, and potentially certain types of cancer (46). Therefore, understanding and manipulating the gut microbiome has become an important direction in modern medical research, aimed at preventing and treating these diseases by regulating the gut microbiota, and our study provides a more precise direction for the prevention and treatment of CG.

The three gut microbiota identified in this study as having a significant impact on CG also play evident roles in other gastrointestinal diseases. Research indicates that changes in the PWY.6708 pathway are associated with gastrointestinal diseases such as inflammatory bowel disease (IBD) and GC (47). Its regulatory imbalance may lead to increased cellular oxidative stress and inflammatory responses (48). *Odoribacter* bacteria are anaerobes in the gut that primarily produce short-chain fatty acids (SCFAs) (49). Studies have shown that the abundance of *Odoribacter* significantly decreases in patients with inflammatory bowel diseases (such as Crohn’s disease and ulcerative colitis), suggesting its potential protective role in maintaining gut health (50). *Coprococcus* bacteria are also major producers of SCFAs, particularly butyrate. Research indicates that a reduction in *Coprococcus* is associated with the occurrence of IBD, irritable bowel syndrome (IBS), and colorectal cancer (51). These bacteria play crucial roles in regulating the gut environment and host immune responses, and their reduction may lead to impaired gut barrier function and chronic inflammation (52).

Metabolites are various small molecule compounds produced or consumed in the metabolic processes of the human body (24). These compounds can be intermediates or end products of metabolic pathways, or substances necessary to sustain life activities. Metabolites encompass various chemical categories, including amino acids, fatty acids, sugars, etc. Research by Pingping Zhou et al. (53) including a Spearman analysis showed that disrupted gut microbiomes are closely related to changes in several related metabolites. Therefore, one reason for the increased concentration and abundance of metabolites in the feces of patients with CG may be the occurrence of dysbiosis within the gut. Research indicates that alterations in gut microbiota may influence amino acid utilization, and gut bacteria can also affect the host’s health by regulating amino acids (54, 55). More and more studies are verifying that metabolites associated with gut microbiota, when influenced by pharmaceutical interventions, play crucial roles in regulating tissue functions and enhancing health (31, 32). Additionally, an imbalance in the gut microbiota and inflammation together cause mucosal damage, disrupting the generation and consumption of metabolites (56). Thus, in MR analysis, four metabolites mediate CG through the disruption of the gut microbiota.

This study found that specific gut microbiota and blood metabolites play important roles in the occurrence and development of CG. These findings provide new perspectives for the diagnosis, treatment, and prevention of CG. By detecting 4-hydroxyphenylacetate levels, palmitoleate (16:1n7) levels, and the phosphate to alanine ratio in the blood, the risk of CG can be identified earlier, enabling early intervention. Additionally, personalized treatment plans (such as dietary adjustments, probiotics, or antibiotics) based on patients’ microbiome and metabolite profiles become possible, improving their microbiome structure. Thus, further understanding the role of specific gut microbiota in CG helps develop new preventive measures, such as prebiotic and probiotic supplements, to maintain a healthy gut microbiome.

Meanwhile, the identified gut microbiota and blood metabolites may influence the development of CG through various mechanisms. For example, *Odoribacter* from the Porphyromonadaceae family and *Coprococcus* from the Lachnospiraceae family may affect gastric inflammation by modulating the immune response of the intestinal mucosa. These microbes can produce short-chain fatty acids (such as butyrate) to regulate intestinal barrier function and immune cell activity (57, 58). Metabolites like 4-hydroxyphenylacetic levels and palmitoleate (16:1n7) levels can influence gastric inflammation through multiple pathways (59). 4-Hydroxyphenylacetic acid has been shown to have potential antioxidant and anti-inflammatory effects, while palmitoleic acid may affect the pathological process of CG by regulating immune responses and metabolic balance (60–62). Furthermore, the gut microbiome can influence gastric neural and hormonal signals through the gut-brain-stomach axis, regulating gastric acid secretion and gastrointestinal motility, thereby further affecting the occurrence and development of CG (63).

Our research offers multiple benefits. First, it pioneers MR analysis to examine the interaction between gut microbiota and CG risk and to assess if metabolites mediate the influence of gut microbiota on CG risk. Second, we have ensured a reliable and effective number of authorizations for the publicly available GWAS data sets. Last, MR analysis is time-efficient and cost-effective relative to the extensive duration required for randomized controlled trials (RCTs), and it adeptly avoids the potential confounders typical of RCTs. Therefore, our study deepens the understanding of the roles of gut microbiota and blood metabolites in CG and highlights the efficiency and effectiveness of MR analysis in clinical research.

### Research limitations

Nonetheless, this study possesses certain inherent limitations that must be acknowledged. First, our analysis was conducted among Europeans and Japanese due to geographic limitations, which might cause biased outcomes due to the restrictions of the GWAS data set and limit its generalizability (64). Second, a single statistical significance threshold parameter (1 × 10^−5^) was used, potentially resulting in the loss of genetic liability variability across samples. Third, these findings have not yet been further validated. Future research will explore these unidentified mediating factors by increasing the sample size and diversity, conducting functional experiments, and performing long-term follow-up studies using multi-omics approaches. Last, environmental factors and dietary habits significantly influence gut microbiota and CG, so more relevant confounders should be considered.

### Conclusion

In summary, our research revealed the causal relationships between three gut microbiota and four blood metabolites with CG and explained the mechanism by which the gut microbiome influences CG through blood metabolites. We recommend paying more attention to CG patients with dysbiosis of the gut microbiome and developing new diagnostic and therapeutic methods by identifying key microbiota and metabolites to improve the prognosis of CG patients. In the future, we plan to increase the sample size and use multi-omics approaches to systematically analyze the interactions between gut microbiota, blood metabolites, and CG, exploring potential unidentified mediating factors.

## Data Availability

The analysis in this study utilized publicly available datasets. The data can be accessed at the following links: (i) Gut microbiota data from the NHGRI-EBI GWAS Catalog at http://ftp.ebi.ac.uk/pub/databases/gwas/summary_statistics/GCST90027001-GCST90028000/. The accession numbers for the gut microbiome data are GCST90027446-GCST90027857. (ii) Chronic gastritis data from the GWAS Catalog (https://gwas.mrcieu.ac.uk/datasets/ebi-a-GCST90018825/). (iii) Blood metabolites data from the GWAS Catalog. Accession numbers for European GWAS: GCST90199621-90201020; accession numbers for non-European GWAS: GCST90201021-90204063.

## SUPPLEMENTAL MATERIAL

Table S1 and S2 spectrum.01490-24-s0001.pdf Table S1: Correspondence table of Gut microbiota. Table S2: Correspondence table of blood metabolites.

ASM does not own the copyrights to Supplemental Material that may be linked to, or accessed through, an article. The authors have granted ASM a non-exclusive, world-wide license to publish the Supplemental Material files. Please contact the corresponding author directly for reuse.
