# Supplementary material for: Role of blood metabolites in mediating the effect of gut microbiota on chronic gastritis
Source: Microbiol Spectr. 2024 Oct 15;12(11):e01490-24. doi: 10.1128/spectrum.01490-24 (PMC11537017; doi:10.1128/spectrum.01490-24)
Supplement: Table S1 and S2 — Table S1: Correspondence table of Gut microbiota. Table S2: Correspondence table of blood metabolites. [file spectrum.01490-24-s0001.pdf]

Table S1 Correspondence table of Gut microbiota

ID	Name
GCST90027458	CRNFORCAT.PWY..creatinine.degradation.I
GCST90027570	PWY.6125..superpathway.of.guanosine.nucleotides.de.novo.biosynthesis.II
GCST90027596	PWY.6708..ubiquinol.8.biosynthesis..prokaryotic.
GCST90027600	PWY.6891..thiazole.biosynthesis.II..Bacillus.
GCST90027635	PWY_REDCITCYC..TCA.cycle.VIII..helicobacter.
GCST90027662	k_Bacteria.p_Actinobacteria.c_Actinobacteria.o_Bifidobacteriales.f_Bifidobacteriaceae
GCST90027688	k_Bacteria.p_Actinobacteria.c_Actinobacteria.o_Bifidobacteriales.f_Bifidobacteriaceae.g_Bifidobacterium
GCST90027692	k_Bacteria.p_Actinobacteria.c_Actinobacteria.o_Coriobacteriales.f_Coriobacteriaceae.g_Gordonibacter
GCST90027697	k_Bacteria.p_Bacteroidetes.c_Bacteroidia.o_Bacteroidales.f_Porphyromonadaceae.g_Odoribacter
GCST90027733	k_Bacteria.p_Proteobacteria.c_Gammaproteobacteria.o_Pasteurellales.f_Pasteurellaceae.g_Haemophilus
GCST90027736	k_Bacteria.p_Actinobacteria.c_Actinobacteria.o_Bifidobacteriales
GCST90027761	k_Bacteria.p_Actinobacteria.c_Actinobacteria.o_Coriobacteriales.f_Coriobacteriaceae.g_Gordonibacter.s_Gordonibacter_pamelaeae
GCST90027800	k_Bacteria.p_Firmicutes.c_Clostridia.o_Clostridiales.f_Ruminococcaceae.g_Ruminococcus.s_Ruminococcus_callidus
GCST90027828	k_Bacteria.p_Bacteroidetes.c_Bacteroidia.o_Bacteroidales.f_Bacteroidaceae.g_Bacteroides.s_Bacteroides_fragilis
GCST90027839	k_Bacteria.p_Bacteroidetes.c_Bacteroidia.o_Bacteroidales.f_Bacteroidaceae.g_Bacteroides.s_Bacteroides_xylanisolvens
GCST90027845	k_Bacteria.p_Firmicutes.c_Clostridia.o_Clostridiales.f_Lachnospiraceae.g_Coprococcus.s_Coprococcus_sp_ART55_1

Table S2 Correspondence table of blood metabolites

ID	Name
GCST90199783	5-methyluridine (ribothymidine) levels
GCST90199965	Fructosyllysine levels
GCST90200338	4-hydroxyphenylacetate levels
GCST90200355	Adenosine 5'-diphosphate (ADP) levels
GCST90200388	Palmitoleate (16:1n7) levels
GCST90200518	X-12839 levels
GCST90200637	X-24531 levels
GCST90200729	3-phosphoglycerate to phosphate ratio
GCST90200765	Phosphate to alanine ratio
GCST90200837	Adenosine 5'-diphosphate (ADP) to glutamate ratio (4556 European)
GCST90200962	Adenosine 5'-diphosphate (ADP) to glutamine ratio (4569 European)
